# Managing remnant cholesterol: role of fenofibrate–statin therapy in reducing triglyceride-rich lipoproteins

**DOI:** 10.3389/fcvm.2026.1837004

**Published:** 2026-06-04

**Authors:** Vikrama Raja, Carlos Aguiar, Nasreen Alsayed, Yogeyaa S. Chibber, Hussein ElBadawi, Michel Farnier, Kumari Pushpa, Lale Tokgözoglu, Alberto Zambon, Jean-Pascal Berrou, Michel P. Hermans

**Affiliations:** 1 Medical Affairs, Abbott Products Operations AG, Basel, Switzerland; 2 Department of Cardiology, Hospital Santa Cruz, Western Lisbon Local Health Unit, Lisbon, Portugal; 3 Department of Endocrinology, Diabetes & Metabolism, Dar Alsaha Medical Center, Manama, Kingdom of Bahrain; 4 Medical Affairs, WNS Global Services, Noida, India; 5 Internal Medicine Department, Wayne State University, Detroit, MI, U.S.A.; 6 Metabolic Unit, My Clinic International, Jeddah, Saudi Arabia; 7 Université Bourgogne Europe, PEC2 UR 7460, Dijon, France; 8 Department of Cardiology, Hacettepe University, Ankara, Turkiye; 9 Department of Medicine, University of Padua, Padua, Italy; 10 Pôle de Recherche Cardiovasculaire, Institut de Recherche Expérimentale et Clinique, Université Catholique de Louvain, Brussels, Belgium

**Keywords:** dyslipidemia, fenofibrate, non-High density lipoprotein cholesterol, remnant cholesterol, residual cardiovascular risk, triglyceride, triglyceride-rich lipoproteins

## Abstract

Dyslipidemia, a key risk factor for atherosclerotic cardiovascular disease (ASCVD), is conventionally managed by lowering low-density lipoprotein cholesterol (LDL-C) level. However, even with on-target LDL-C and optimal control of traditional risk factors such as hypertension and diabetes, a substantial residual risk of major adverse cardiovascular events (MACE) persists. A growing body of evidence suggests that remnant cholesterol (RC) – the cholesterol content of triglyceride-rich lipoproteins (TRLs) – partially contributes to this residual risk. Consequently, non-high-density lipoprotein cholesterol (non-HDL-C) is considered a better measure of the ASCVD risk and a comprehensive treatment goal for dyslipidemia. Statin therapy, the standard care for dyslipidemia management, falls short of mitigating RC-related residual risk. The global rise in obesity, type 2 diabetes, and metabolic syndrome has led to a growing prevalence of hypertriglyceridemia, underscoring the need for adjunctive therapies that target TRLs and lower non-HDL-C levels. Fenofibrate, a well-established TRL-lowering agent, has demonstrated efficacy in reducing non-HDL-C when used in combination with statins. Evidence from clinical trials and real-world studies suggests potential benefits of this combination in reducing cardiovascular risk, particularly in patients with elevated triglyceride levels. Moreover, long-term studies – spanning up to two decades –have affirmed the safety and tolerability of fenofibrate, reinforcing its role as a valuable add-on therapy to address remnant cholesterol and residual cardiovascular risk.

## Graphical Abstract

**Figure:**
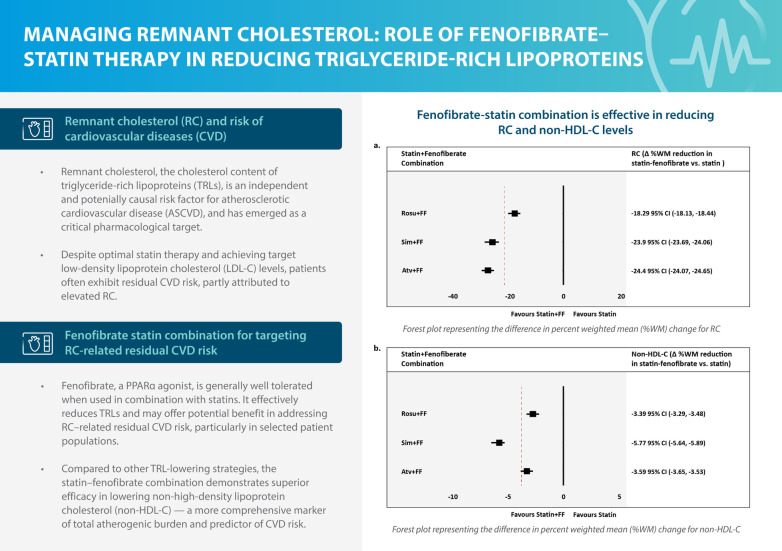
Efficacy of fenofibrate-statin combination therapy in managing remnant cholesterol and CVD risk. Remnant cholesterol (RC), the cholesterol content of triglyceride-rich lipoproteins (TRLs), is an independent risk factor for atherosclerotic cardiovascular disease (ASCVD), and partly contributes to the residual cardiovascular disease (CVD) risk in patients even with at-target low-density lipoprotein cholesterol (LDL-C). Fenofibrate in combination with statins is an efficacious strategy to reduce RC levels in addition to non-high density lipoprotein cholesterol (non-HDL-C) for managing residual CVD risk in statin-treated patients. Rosu + FF, Rosuvastatin and fenofibrate combination; Sim + FF, Simvastatin and fenofibrate combination; Atv + FF, Atorvastatin and fenofibrate combination; %WM, percent weighted mean.

## Highlights

- Remnant cholesterol (RC), the cholesterol content of triglyceride-rich lipoproteins (TRLs), is an independent and potentially causal risk factor for atherosclerotic cardiovascular disease (ASCVD) that has emerged as a critical pharmacological target.
- Despite optimal statin therapy and achieving target low-density lipoprotein cholesterol (LDL-C) levels, patients often exhibit residual cardiovascular disease (CVD) risk, partly attributed to elevated RC.
- Fenofibrate, a PPAR*α* agonist, is generally well tolerated when used in combination with statins. It effectively reduces TRLs and may offer potential benefit in addressing RC–related residual CVD risk, particularly in selected patient populations.
- Compared to other TRL-lowering strategies, the statin–fenofibrate combination demonstrates superior efficacy in lowering non-high-density lipoprotein cholesterol (non-HDL-C) – a more comprehensive marker of total atherogenic burden and predictor of CVD risk.

## 1 Introduction

Atherosclerotic cardiovascular diseases (ASCVD) remain the leading cause of mortality globally, despite notable advances in pharmacological interventions (1). Among the modifiable risk factors, dyslipidemia plays a central role in the development and progression of ASCVD, alongside hypertension, diabetes, and obesity (2). Current lipid-lowering therapies—primarily statins, or 3-hydroxy-3-methylglutaryl coenzyme A (HMG-CoA) reductase inhibitors—serve as the first-line treatment for managing dyslipidemia by effectively reducing low-density lipoprotein cholesterol (LDL-C) (3–7).

However, even in patients achieving recommended LDL-C targets and having other traditional cardiovascular risk factors under optimal control, a substantial risk of major adverse cardiovascular events (MACE) – referred to as residual cardiovascular disease (CVD) risk – persists, prompting investigation into treatment strategies that can address this risk (2, 8).

Accumulating evidence underscores the importance of non-high-density lipoprotein cholesterol (non-HDL-C) as a predictor of ASCVD risk. Calculated by subtracting high-density lipoprotein cholesterol (HDL-C) from total cholesterol (TC), non-HDL-C encompasses the cholesterol content of all atherogenic lipoproteins (9). Studies have shown that in statin-treated individuals with on-target LDL-C, but discordant (above target) non-HDL-C, the risk of myocardial infarction (MI) increases significantly—hazard ratio (HR) 1.78; [95% confidence interval (CI): 1.35 to 2.34] (10). Apolipoprotein B100 (ApoB), the principal apolipoprotein associated with all atherogenic lipoproteins, provides an effective measure of all atherogenic particles in blood. Similar to non-HDL-C, discordant (elevated) ApoB levels in patients with on-target LDL-C significantly increase the risk of MI and all-cause mortality as compared to patients with concordant ApoB and LDL-C levels (10).

These observations highlight the limitations of lipid-lowering strategies that focus exclusively on LDL-C and underscore the need to address residual cardiovascular risk more comprehensively. Accordingly, both non–HDL-C and ApoB are increasingly recognized as superior markers of residual risk and as more appropriate therapeutic targets in dyslipidemia. The 2019 ESC/EAS recommendations for ApoB and non-HDL-C targets in very high, high, and moderate risk patients are <65, <80, and <100 mg/dL and <85, <100, and <130 mg/dL, respectively (4). In the context of residual CVD risk, clinical strategies should aim for a reduction in non-HDL-C and ApoB, rather than LDL-C alone.

All ApoB-associated serum lipoproteins <70 nm in size are considered atherogenic. Besides LDLs (including Lp[a], the other major ApoB-associated atherogenic lipoproteins are the triglyceride-rich lipoproteins (TRLs), which include the very low-density lipoproteins (VLDLs), intermediate-density lipoproteins (IDLs), chylomicron remnants, and their respective metabolic intermediates (9, 11). The cholesterol content of TRLs is referred to as remnant cholesterol (RC). Operationally, RC represents the non-LDL-C fraction of non-HDL-C and can be calculated as total cholesterol (TC) minus LDL-C minus HDL-C. Due to its close relationship with serum TG, RC may be approximated as TGs in mg/dL divided by 5 (TG in mmol/L divided by 2.2) (11, 12). For clarity and consistency, the primary definition of RC adopted in this review is the total cholesterol content of all the TRLs.

Mounting evidence suggests that RC is a key contributor to CVD risk, as documented in the next section, and is considered a clinically relevant pharmacological target for interventions aimed at reducing residual risk in patients who are otherwise well-managed for LDL-C and other conventional CVD risk factors.

## 2 Remnant cholesterol and CVD risk

Large epidemiological studies have shown a positive association of RC with CVD risk, and several Mendelian randomization studies have pointed to a potential causal link between elevated RC and CVD (13–15). Importantly, multiple studies have confirmed the role of RC in atherosclerotic plaque formation and progression (8, 16).

### 2.1 RC and risk of ASCVD in the primary prevention cohort

The association of TRLs and their remnants with future CVD risk in primary prevention cohorts has been unequivocally demonstrated by several epidemiological studies and was recently reviewed by Li et al., 2025 (8). For each 1-standard deviation (1-SD) rise in RC, a 23% rise in coronary heart disease (CHD) risk is predicted, even after adjusting for established cardiovascular risk factors (HR 1.23; 95% CI: 1.06–1.42, *P* *<* 0.01) (17). Large prospective cohort studies with several decades of follow-up have found a significant positive association between RC and ASCVD risk (HR 1.65; 95% CI: 1.45–1.89), even after multivariate adjustments for LDL-C (17), and a linear relationship with all-cause mortality (18). Higher levels of RC >39 mg/dL (>1 mmol/L) – prevalent in about 22% of the general population – were found to be associated with a two-fold higher risk of ASCVD and death (13).

### 2.2 RC and residual risk of ASCVD in patients on lipid-lowering therapy

RC is also a strong predictor of residual CVD risk in statin-treated patients with controlled LDL-C levels (19). A study on statin-treated patients with coronary artery disease (CAD) on lipid-lowering therapies and LDL-C levels <100 mg/dL, reported RC as a better predictor of MACE and underscored the importance of targeting RC to lower the residual risk (20). Another study assessing the risk of recurrent adverse CV events in statin-treated patients after acute coronary syndrome found that high levels of RC was a significant risk factor for secondary events, independent of conventional risk factors (HR 2.94; 95% CI: 1.40–6.18; *P* < 0.01) (21). Elevated RC levels (>25 mg/dL) have been observed to result in atherosclerotic plaque progression among both statin- and proprotein convertase subtilisin/kexin type 9 (PCSK9)-treated patients (22). Consequently, raised levels of RC are associated with all-cause mortality in patients with coronary heart disease (CHD), contributing to 8%–18% of the residual risk of mortality (23).

### 2.3 Role of RC in ASCVD risk among high-risk populations

The prevalence of diabetes, obesity, and metabolic syndrome is rising globally (24). One common characteristic of these chronic ailments is the presence of high levels of TRLs and TRL remnants (25, 26). Overall, 10%–20% of the general population and 15% of statin-treated patients are estimated to have high TG/TRL levels (27, 28). Among ASCVD patients who have achieved target LDL-C levels, around 25% are still hypertriglyceridemic (29). Furthermore, up to 30% of individuals with type 2 diabetes (T2D) exhibit a dyslipidemic profile characterized by high triglycerides and low HDL-C levels—placing them at increased cardiovascular risk (30).

In patients with diabetes and obesity, every 10 mg/dL rise in RC levels reportedly leads to a 21% increase in MACE, and >30 mg/dL RC levels are considered at a high risk of CVD, irrespective of LDL-C levels (14). About one in five ASCVD events in patients with diabetes is attributed to elevated RC (31). Elevated RC levels have also been shown to be significantly associated with young-onset of acute myocardial infarctions (AMI) in patients with T2D (OR: 1.415; 95% CI 1.189–1.684; *P* < 0.001), even after adjusting for traditional CV risk factors (16).

These studies underscore the independent, potentially causal role of RC and as a predictor of ASCVD, highlighting the critical need to address RC-related residual CV risk, particularly in high-risk individuals and those undergoing secondary prevention.

## 3 RC-related residual CV risk – an unmet need

International guidelines for the management of dyslipidemia and CVD – including those from the European Society of Cardiology (ESC 2019, 2023, 2025), Americal Heart Association/ American College of Cardiology (AHA/ACC 2018, 2023), and American Association of Clinical Endocrinology (AACE 2025) – recognize TRLs and RC as potent atherogens, particularly in patients with T2D, obesity, and other metabolic disorders. These guidelines emphasize the need for strategies aimed at lowering TG and RC levels. In addition, they endorse non-HDL-C and ApoB as more comprehensive markers of atherogenic burden, beyond LDL-C, and as appropriate secondary targets in lipid-lowering therapy (3, 4, 6, 7, 32, 33).

While statins remain the first-line treatment for managing mild-to-moderate hypertriglyceridemia (150–499 mg/dL), their efficacy in lowering LDL-C does not fully mitigate the residual risk driven by elevated TG and RC (3, 4). The guideline-recommended use of long-chain omega-3 fatty acids like eicosapentaenoic acid (EPA), as add-on therapy to statins for the management of hypertriglyceridemia, remains contentious, as evidence from large-scale trials has not consistently demonstrated a definitive benefit in reducing RC-mediated residual CV risk (4, 32).

The Study to Assess Statin Residual Risk with Epanova in High Cardiovascular Risk Patients with Hypertriglyceridemia (STRENGTH), which evaluated a combination of eicosapentaenoic acid and docosahexaenoic acid (EPA + DHA), failed to demonstrate cardiovascular benefit, leading to the disengagement of EPA + DHA combinations in recent guidelines (34). In contrast, the Reduction of Cardiovascular Events with Icosapent Ethyl–Intervention Trial (REDUCE-IT), which evaluated Icosapent Ethyl (IPE)—a purified EPA formulation—reported a 25% relative risk reduction in major cardiovascular events (HR 0.75; *P* < 0.001), along with modest reductions in ApoB (−2.5% vs 7.8%; *p* < 0.001) and non-HDL-C (−3.1% vs 9.8%; *P* < 0.001) (35).

The discordant findings between the two trials may be attributable to differences in study design. REDUCE-IT used a higher dose of purified EPA (3840 mg/day), achieving plasma EPA levels of 144.0 μg/mL at 12 months, while STRENGTH participants received 2,200 mg of EPA daily (in combination with 800 mg of DHA), resulting in a lower plasma EPA concentration of 89.6 μg/mL Differences in EPA dosing and achieved plasma EPA levels are reported to be directly proportional to the clinical benefit. Another critical and more debated distinction is the choice of comparator oil – corn oil in STRENGTH versus mineral oil in REDUCE-IT. The use of mineral oil has raised concerns regarding its potential pro-inflammatory effects and adverse impacts on lipid parameters, as evidenced by increases in ApoB, LDL-C, and hsCRP in the placebo arm of REDUCE-IT. This may have contributed, at least in part, to the magnitude of benefit observed in REDUCE-IT relative to STRENGTH (36, 37).

Notably, the IPE-led CVD risk reduction in REDUCE-IT appeared to be independent of changes in TG or TRL levels, suggesting alternative mechanisms such as anti-inflammatory or plaque-stabilizing effects may be responsible for the observed benefits (35). Besides, the Randomized Trial for Evaluation in Secondary Prevention Efficacy of Combination Therapy–Statin and Eicosapentaenoic Acid (RESPECT-EPA) using IPE as statin add-on treatment failed to show statistically significant CV benefits in patients with coronary artery disease (38). Consequently, the management of hypertriglyceridemia and RC–related residual risk remains a clinical challenge, with limited evidence-based guidance and uncertainty around optimal therapeutic strategies (3, 4, 32).

This necessitates expanding the therapeutic arsenal beyond LDL-C-lowering strategies and calls for re-evaluating RC-targeted therapies that can complement statin treatment, enabling a more comprehensive reduction in non-HDL-C levels. Such statin add-on therapies will offer a more holistic lipid-lowering approach that addresses both LDL-C and RC-driven residual risk.

## 4 Fenofibrate has an established role in lowering TRLs

**Figure 1:**
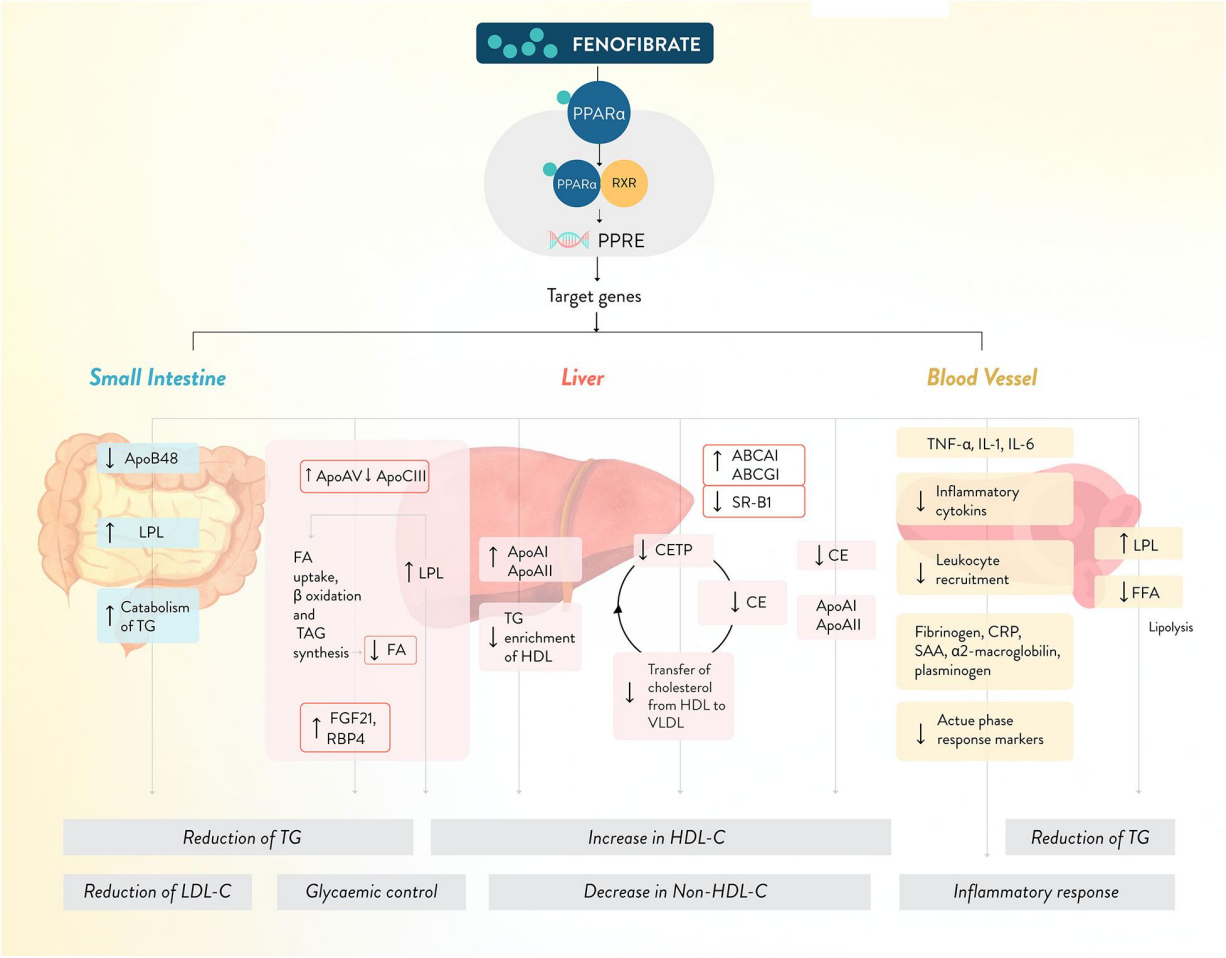
**Mechanism of action of fenofibrate in atherogenic dyslipidemia:** fenofibrate is a fibrate derivative that acts as a PPAR*α* agonist. By binding to PPAR*α*, it activates a specific cascade of genes that regulate fatty acid metabolism in the liver, adipose tissue, and endothelial cells. The figure describes the target genes activated or suppressed by activating PPAR*α* in these tissue types. PPAR*α* manifests the expression of the LPL, apolipoproteins (*APOAV*), and growth factors (*FGF21*) while downregulating the expression of *APOCIII* leading to a reduction in TG levels. Further, it positively stimulates the expression of apolipoproteins (*APOAI*, and *APOAII*), and lipid transporters (*ABCAI & ABCAII*) leading to an increase in HDL. The reduction in TG and increase in HDL cutbacks the residual risk of atherosclerotic cardiovascular diseases. The figure shows the immunomodulatory property of fenofibrate - independent of its role on lipids. Fenofibrate additionally down-regulates the expression of other genes involved in inflammatory response such as acute phase response markers (*CRP*, fibrinogen, *SAA*) and inflammatory cytokines (*TNF, IL*). ApoAI, apolipoprotein A-I; ApoAII, apolipoprotein A-II; ApoB, Apolipoprotein B; ApoAV, Apolipoprotein A-V; ApoCIII, Apolipoprotein C-III; CETP, cholesteryl ester transfer protein; CE, Cholesterol efflux; CRP, C-reactive protein; FFA, Free fatty acids; FGF21, Fibroblast growth factor 21; RBP4, Retinol binding protein 4; HDL, High-density lipoprotein; IL, Interleukin; LDL, Low-density lipoprotein; LPL, Lipoprotein lipase; PPAR, Peroxisome proliferator-activated receptor; RXR, Retinoid X receptor; SAA, Serum amyloid A; TG, Triglycerides; TNF, Tumor Necrosis Factor; VLDL, Very low-density lipoprotein.

Fenofibrate, a fibrate-class drug, functions as a peroxisome proliferator-activated receptor *α* (PPAR*α*) agonist and is well-established for its TG/TRL-lowering properties (39). PPARs (*α*; *β*/*δ*; and *γ* subtypes) are nuclear receptors involved in the adaptive response to fasting by regulating energy homeostasis (40, 41)^.^ Upon ligand binding, PPAR*α* undergoes conformational changes, recruits co-activators, and dimerizes with the retinoid X receptor (RXR). This activated PPAR*α*/RXR complex binds to PPAR response elements (PPREs), modulating the expression of multiple genes involved in lipid metabolism (transport, *β*-oxidation, ketogenesis), fat storage, and glucose homeostasis across tissues such as the liver, gut, and endothelium (Figure 1) (42).

### 4.1 The role of fenofibrate in regulating plasma lipids

Fenofibrate, as a PPAR*α* activator, regulates the expression of a host of lipid modulatory genes such as lipoprotein lipase (*LPL*), apolipoproteins (*APOAI*, *APOAII*, *APOAV*, *APOCIII*), lipid transporters (*ABCAI* & *ABCAII*), angiopoietin-like protein 4, as well as genes involved in fatty acid metabolism (Acyl CoA synthase) (40). Fenofibrate can regulate plasma lipids through 1) higher lipolysis of TRLs and their remnants, 2) enhanced uptake of fatty acids and lower production of TG by the liver, 3) enhanced clearance of LDL particles by LDL receptors, and 4) increased HDL numbers and reverse cholesterol transport (39, 43).

Fenofibrate is suggested to enhance the lipolysis of triglyceride-rich lipoproteins (TRLs) through increased lipoprotein lipase (LPL) activity and improved TRL accessibility, attributed to reduced apoC-III content. Additionally, fenofibrate inhibits hepatic TG production by downregulating fatty acid synthesis and reducing fatty acid availability in the liver, primarily through the upregulation of fatty acid transporter proteins and activation of the *β*-oxidation pathway (39). In addition to TRLs, fenofibrate also enhances LDL-C clearance and reduces the number of small dense LDL particles (39, 44).

### 4.2 The role of fenofibrate in chronic inflammation

Fenofibrate exhibits PPAR*α*-mediated anti-inflammatory effects through both direct and indirect mechanisms. It is suggested to repress inflammation indirectly through improved lipid metabolism and restoring lipid homeostasis (45). Additionally, clinical studies have demonstrated a direct effect of fenofibrate on inflammatory pathways independent of its role in lipid and glucose metabolism, with a marked decrease in systemic inflammatory markers such as C-reactive protein and Interleukin 6, in patients with metabolic syndrome (46). It also modulates key inflammatory pathways related to nuclear factor kappa-B, sirtuin 1, toll-like receptor 4, adenosine mitogen-activated protein kinase, and interleukins such as Interleukin 1 (45).

## 5 Fenofibrate statin combination for targeting RC-related residual risk

**Figure 2:**
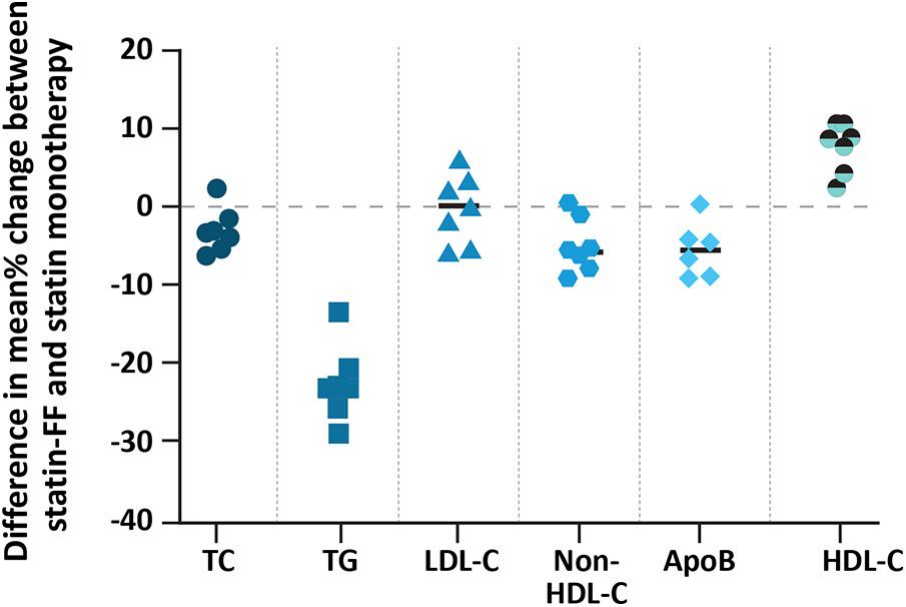
**Fenofibrate is efficacious in improving serum lipid parameters as combinatorial therapy with statin**. The scatter plot represents the percent change in lipid parameters from the baseline in fenofibrate and statin combination therapy as compared to statin monotherapy from different studies; HDL,C, High-density lipoprotein cholesterol; LDL-C, low-density lipoprotein cholesterol; TG, Triglycerides; TC, total cholesterol; ApoB, Apolipoprotein B; noN,HDL,C, non-high-density lipoprotein cholesterol Studies considered for this scatter plot are: ACCORD (48); SAFARI (47); Jones et al., 2009 (80); Goldberg et al., 2009 (81); Roth et al., 2010 (82); Farnier et al., 2010 (83). The description of the studies and values for lipid parameters depicted in the graph are presented in detail in Supplementary Table S1.

Efficacy studies have demonstrated the benefits of adding fenofibrate to standard statin therapy for improving lipid parameters. One of the first studies examining the fenofibrate-statin combination, as compared to simvastatin monotherapy, was the SAFARI trial. The study reported a substantial reduction in TRL levels (TG: 23.6%, VLDL-C: 25%; *P* ≤ 0.001) with modest benefits on LDL-C (5.4%) (*P* ≤ 0.001) in addition to significant lowering of non-HDL-C (9.2%, *P* ≤ 0.001) and ApoB (9.8%, *P* ≤ 0.001) levels (47). Similar results have been reported by numerous studies using both high- and moderate-intensity statins, demonstrating an overall reduction in atherogenic lipoproteins (Figure 2 and Supplementary Table S1).

**Figure 3:**
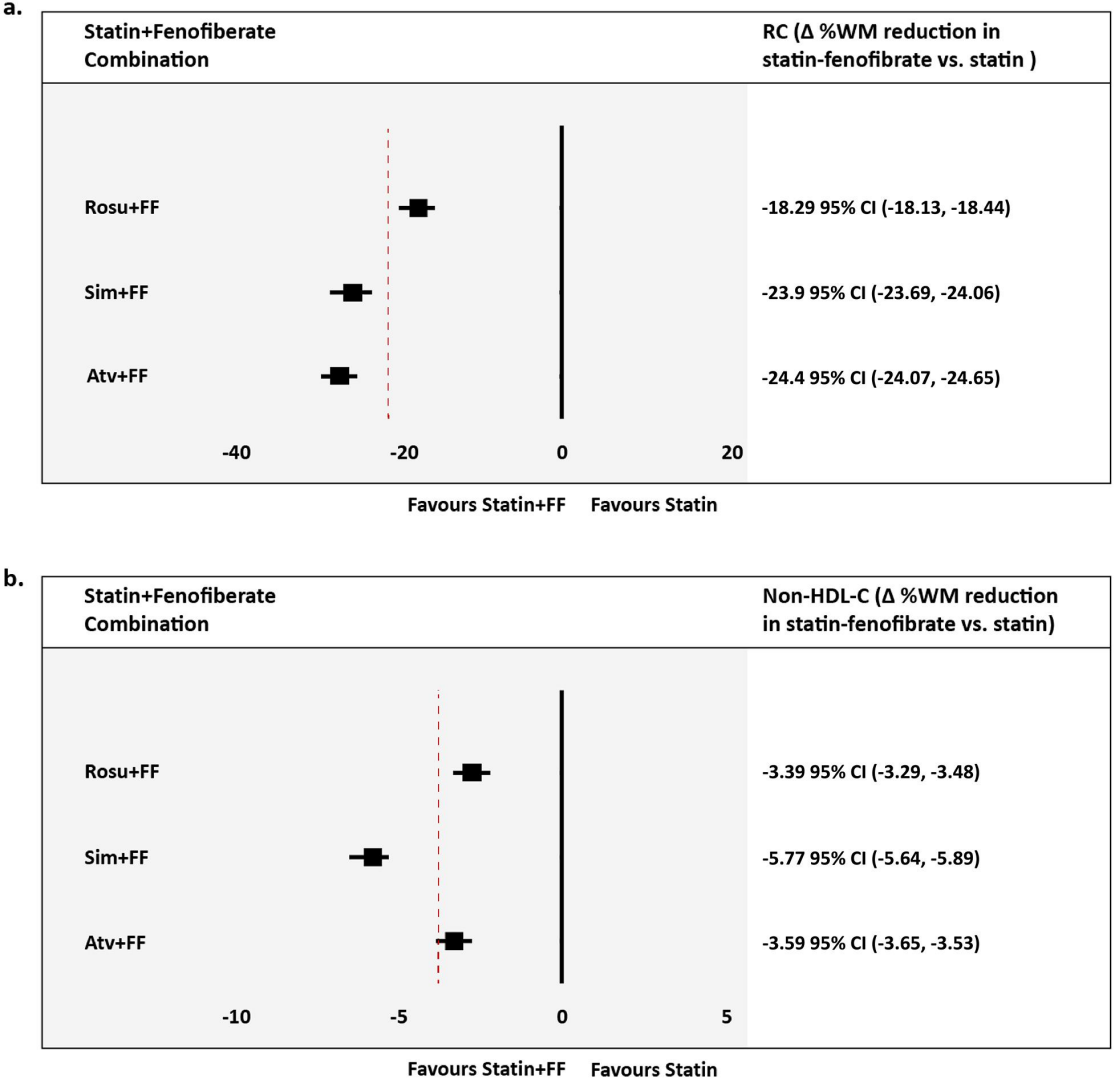
**Fenofibrate–statin combination is more efficacious in reducing RC and noN,HDL,C levels**. The Forest plots represent the difference in percent weighted mean change for RC **(a)** or non-HDL-C **(b)** levels in fenofibrate-statin combination and statin monotherapy. Atv + FF, Atorvastatin + Fenofibrate; Sim + FF, Simvastatin + Fenofibrate; Rosu + FF, Rosuvastatin + Fenofibrate; %WM, Percent weighted mean. The studies used for this analysis and the method details are described in Supplementary Table S3 and Supplementary Methods S1.

Using a meta-analysis framework, the pooled treatment effects of fenofibrate-statin combination therapy vs. statin monotherapy were evaluated based on studies employing equivalent statin and fenofibrate doses across intervention and comparator arms, thereby enabling direct comparison of treatment effects. Details of the included studies, along with raw lipid values and calculated mean relative changes, are provided in Supplementary Table S3. Pooled estimates of lipid parameters were derived using the methodology outlined in the Supplementary Method S1 with results presented as a forest plot in Figure 3 and Supplementary Figure S1. Across multiple statins, fenofibrate add-on therapy demonstrated a consistent effect in reducing RC, non-HDL-C, and ApoB compared with statin monotherapy.

The benefits of the fenofibrate-statin combination as compared to statin monotherapy in improving remnant cholesterol and non-HDL are complemented with data from a large CVD outcome trial – The Action to Control Cardiovascular Risk in Diabetes (ACCORD) study – which demonstrated that combining fenofibrate with statin therapy may reduce CVD risk, particularly in patients with elevated TRLs at baseline. These findings are further reinforced by multiple real-world observational studies, underscoring the clinical value of fenofibrate add-on therapy in addressing TRL-driven residual CVD risk (Table 1).

**Table 1:** Major clinical trials and real-world studies of the fenofibrate monotherapy and fenofibrate-statin combination therapy.

Study name	Study type	Study population	Study intervention	Study outcomes measured	Result in the overall population	Result in high TG (>200 mg/dL), low HDL-C (<40 mg/dL) subgroup
ACCORD	Randomized controlled trial	Type 2 diabetes	Fenofibrate + Simvastatin vs. Simvastatin + placebo	Non-fatal MI, non-fatal stroke, cardiovascular death	8% reduction (NS)	**32%** [HR 0.67, 95% CI (0.47–0.97)]
FIELD	Randomized controlled trial	Type 2 diabetes	Fenofibrate (19% with statin) vs. Placebo (36% statin)	CHD death or non-fatal MI	11% reduction (NS)	**26%** (*P* = 0.01)
ACCORDION	Post-trial follow-up of ACCORD	Type 2 diabetes	Fenofibrate + Simvastatin vs. Simvastatin + placebo	Non-fatal MI, non-fatal stroke, cardiovascular death	7% reduction (NS)	**27%** (HR 0.73; 95% CI, 0.56–0.95)
ECLIPSE-Real	Propensity score matched cohort	Metabolic syndrome	Fenofibrate + statin vs. Statin monotherapy	CHD, stroke, cardiovascular death	-	**26%** (HR, 0.74; 95% CI, 0.58 to 0.93)
Hong et al., 2024	Propensity score matched cohort	Diabetes with >150 mg/dL TG	Fenofibrate + statin vs. Statin monotherapy	MI, MI/stroke and all-cause mortality	-	MI: **12%** (HR 0.878; 95% CI, 0.827–0.933); All-cause mortality: **28.4%** (HR 0.716; 95% CI, 0.685–0.749)
Kim et al, 2024	Propensity score matched cohort	Metabolic syndrome	Fenofibrate + statin vs. Omega-2 FA + Statin monotherapy	ischaemic heart disease, ischaemic stroke, and cardiovascular death	-	**21%** (HR 0.79; 95% CI 0.74–0.83)

### 5.1 ACCORD – A cardiovascular outcome trial

The Action to Control Cardiovascular Risk in Diabetes (ACCORD) study evaluated the fenofibrate-statin combination in 5,518 patients with T2D for a mean follow-up of 4.7 years (48). The primary outcome was the first occurrence of MACE (a composite of non-fatal MI, stroke, or CVD-related mortality) and the secondary outcomes included MACE combined with revascularization and hospitalization due to congestive heart failure.

No significant differences were reported in the primary or secondary outcomes in the overall population, likely because most patients (> 80%) did not display elevated TRL levels (median TG -144 mg/dL) (49). However, the combination therapy was demonstrated to be particularly beneficial for a subgroup displaying high TG (>200 mg/dL) and low HDL-C (<34 mg/dL) levels, representing 17% (941 out of 5,518 patients) of the total study population (49, 50). In this prespecified subgroup analysis, the fenofibrate-statin combination resulted in a 32% reduction (12.37% vs. 17.32%) of CVD events (HR 0.67; 95% CI 0.47–0.97) as compared to the placebo (50, 51).

The Fenofibrate Intervention and Event Lowering in Diabetes (FIELD) study, a placebo-controlled trial in 9,795 patients with T2D, was another major study evaluating the benefits of fenofibrate monotherapy on CVD risk. Similar to ACCORD, the results for this trial were neutral in the overall population; however, the benefits of fenofibrate treatment were notably higher in the FIELD subgroup with elevated TG (>200 mg/dL) and low HDL-C (<50 mg/dL) levels, resulting in a 26% (*P* = 0.01) relative risk reduction of CVD events compared to the placebo (52, 53).

A five-year extended, observational follow-up of ACCORD-lipid participants in the ACCORD Follow-On Study (ACCORDION) detected a “legacy” effect of fenofibrate add-on treatment to statins. The study reported a 27% (HR 0.73; 95% CI, 0.56–0.95) CVD risk reduction in the mixed dyslipidemia subgroup (*N* = 853) upon fenofibrate-statin treatment compared to placebo during a combined duration of 9 years, including the trial and post-trial periods (54). Furthermore, a secondary analysis of the ACCORDION study also revealed a beneficial legacy effect on all-cause mortality – a 35% reduction (HR 0.65; 95% CI 6–55; *P* = 0.02) – in these mixed dyslipidemia patients who were initially assigned to the fenofibrate-statin add-on therapy (55).

### 5.2 Real-world studies

Evidence from real-world observational cohort studies has further supported the use of the fenofibrate-statin combination to reduce RC-driven residual CVD risk. The Effectiveness of Fenofibrate Therapy in Residual Cardiovascular Risk Reduction in the Real World Setting (ECLIPSE-REAL), a propensity score-matched cohort study in patients with metabolic syndrome, reported 26% (HR, 0.74; 95% CI 0.58–0.93) reduction in MACE among participants receiving fenofibrate-statin therapy (*N* = 2156) as compared to statin monotherapy (*N* = 8549) (56). Similar outcomes were reported by another large propensity score-matched cohort study in patients with diabetes and TG >150 mg/dL. In this primary prevention cohort (*N* = 110,723), addition of fenofibrate to statin therapy led to a 12% reduction in MI (HR 0.878; 95% CI 0.827–0.933) and a marked 28.4% reduction in all-cause mortality (HR 0.716; 95% CI 0.685–0.749) (57). Fenofibrate-statin combination was also associated with lower all-cause mortality (HR 0.826; 95% CI 0.795–0.858) and CVD risk (HR 0.929; 95% CI 0.898–0.962) in the general population, among individuals with high serum levels of TG/TRL levels (>200 mg/dL) (58). Interestingly, another propensity score matched cohort study comparing CV benefits of fenofibrate vs. omega-3 fatty acids as add-on therapy to statins in patients with metabolic syndrome found that fenofibrate combination therapy was associated with a lower risk of MACE (HR 0.79; 95% CI 0.74–0.83). These results were particularly evident in patients receiving a mean daily dose of ≤1–2 g of omega-3 fatty acids. However, the CV risks in patients receiving >2 g/day of omega-3 fatty acids were comparable to the fenofibrate group (HR, 1.13; 95% CI, 0.96–1.33) (59).

Evidence from these studies suggest that fenofibrate in combination with statins can be effective strategy to manage dyslipidemia marked by hypertriglyceridemia to potentially reduce RC-related CVD risk. These findings also suggest that TG levels ≥150–200 mg/dL may indicate residual CVD risk in statin-treated patients, regardless of diabetes status or attainment of LDL-C targets. In such cases – especially when further statin intensification is not feasible – adding fenofibrate may be a beneficial therapeutic option.

### 5.3 Lowering non-HDL-C levels is key to reducing RC-related residual CVD risk

Cardiovascular disease risk reduction with fibrate therapy appears to correlate directly with the extent of non-HDL-C reduction rather than TG lowering. This association suggests that the beneficial effects of fenofibrate on residual ASCVD risk are largely mediated through its ability to lower atherogenic lipoproteins, as reflected by non-HDL-C and ApoB levels (4, 60).

This hypothesis was supported by the findings of the Pemafibrate to Reduce Cardiovascular Outcomes by Reducing Triglycerides in Patients with Diabetes (PROMINENT) trial, which evaluated pemafibrate – a selective PPAR*α* modulator – in statin-treated patients with type 2 diabetes and mild-to-moderate hypertriglyceridemia. Despite achieving significant reductions in triglycerides (26%; 95% CI: −28.4 to −24.10) and remnant cholesterol (25.6%; 95% CI: −27.3 to −24.0), pemafibrate did not reduce residual CVD risk. This neutral outcome is indicated as attributable, at least partially, to its failure in lowering non-HDL-C (0.2%; 95% CI: −1.3 to 1.0) and increase in ApoB (4.8%; 95% CI: 3.8 to 5.8) and LDL-C levels (12.3%; 95% CI: 10.7 to 14.0) (61, 62).

In contrast, the fenofibrate-statin combination has demonstrated reductions in total atherogenic lipoproteins, as evidenced by decreases in non-HDL-C and ApoB, in addition to TG/TRL levels (Figure 3 and Supplementary Figure S1). These findings support the premise that comprehensive lipid modulation – as reflected by the reduction of non-HDL-C and ApoB – is critical for effectively lowering RC-related residual cardiovascular risk.

Consistent with this evidence, the international dyslipidemia guidelines, such as ESC/EAS 2025 and 2019, do not recommend routine use of fenofibrate–statin combination therapy for all patients but suggest that it may be considered in selected high-risk individuals for primary prevention who have achieved LDL-C targets but continue to exhibit persistent hypertriglyceridemia [TG >200 mg/dL (2.3 mmol/L)]. Given the limited and heterogeneous evidence for incremental cardiovascular benefit with combination therapy, these guidelines emphasize individualized decision-making and highlight the need for further studies to better define the patient populations most likely to benefit (4, 33).

## 6 Fenofibrate-statin combination – beyond lipid-lowering effects

Besides being at higher risk for CVD, patients with diabetes are also at high risk of developing microvascular complications affecting the eyes, kidneys, and nervous system, causing diabetic retinopathy (DR), nephropathy, and neuropathy, respectively. As per estimates, about 25%–50% of diabetic patients are diagnosed with microvascular complications resulting in end-stage renal disease (ESRD), blindness, foot ulcerations, and non-traumatic amputations (63).

Combining fenofibrate with standard statin therapy to reduce residual CVD risk could lead to an additional benefit of lowering/delaying various microvascular complications in patients with diabetes. Data from the ACCORD, FIELD, and ECLIPSE-REAL studies demonstrated, respectively, 40% (*p* = 0.006), 34% (*p* = 0.022), and 12% (*p* = 0.005) reduction in DR progression upon fenofibrate-statin treatment as compared to statin monotherapy (52, 64, 65). In the ECLIPSE-REAL study, DR benefits were observed in patients with pre-existing DR, in whom it lowered the risks of vitreous haemorrhage (HR 0.86, *P* = 0.042), laser photocoagulation (HR 0.86, *P* = 0.009), and intraocular injection therapy (HR 0.73, *P* = 0.003) (65). Conclusive evidence of fenofibrate's benefits in DR progression came from the Lowering Events in Non-proliferative Retinopathy in Scotland (LENS) study. This double-blinded, placebo-controlled trial was specifically designed to investigate the ocular effects of fenofibrate and had ∼75% of the participants on background statin therapy. Treatment with fenofibrate (vs. placebo) led to a 27% reduction (HR 0.73; 95% CI, 0.58–0.91) in DR progression and a 50% lower (HR 0.50; 95% CI, 0.30–0.84) risk of macular oedema. Furthermore, these benefits were consistent across age, diabetes types, disease severity, and renal function, showing broad effectiveness of fenofibrate therapy in slowing diabetic retinopathy progression (66). Taking cognizance of the firm evidence of fenofibrate therapy in retarding DR progression, several countries, including Australia and Singapore, have approved the use of fenofibrate for DR treatment (67).

Fenofibrate therapy is also associated with other microvascular benefits. The ACCORD study reported significantly fewer events of microalbuminuria [[38.2 vs. 41.6% (*P* = 0.01)] and macroalbuminuria [10.5 vs. 12.3% (*P* = 0.04)] in the combination treatment arm (48). The FIELD study also reported similar renal benefits, a 14% reduction in the proportion of patients showing progression to albuminuria and an increase by 15% in those showing regression of albuminuria (*P* < 0.002). The FIELD study also reported a marked reduction (37%; *P* = 0.011) in the total number of non-traumatic lower limb amputations from peripheral artery disease in the fenofibrate treatment arm compared to the control (52, 53, 68). These microvascular benefits, supported by data from *in vitro* analysis, suggest a favorable role for fenofibrate in promoting vessel repair and capillary protection against damage or leakage (53). These findings are particularly encouraging, as they indicate that fenofibrate, when used in combination with statin therapy, may offer added benefits in mitigating microvascular complications—beyond its well-established macrovascular effects—which appear to be independent of its lipid-modifying properties (69).

## 7 The safety profile of fenofibrate-statin combination

**Figure 4:**
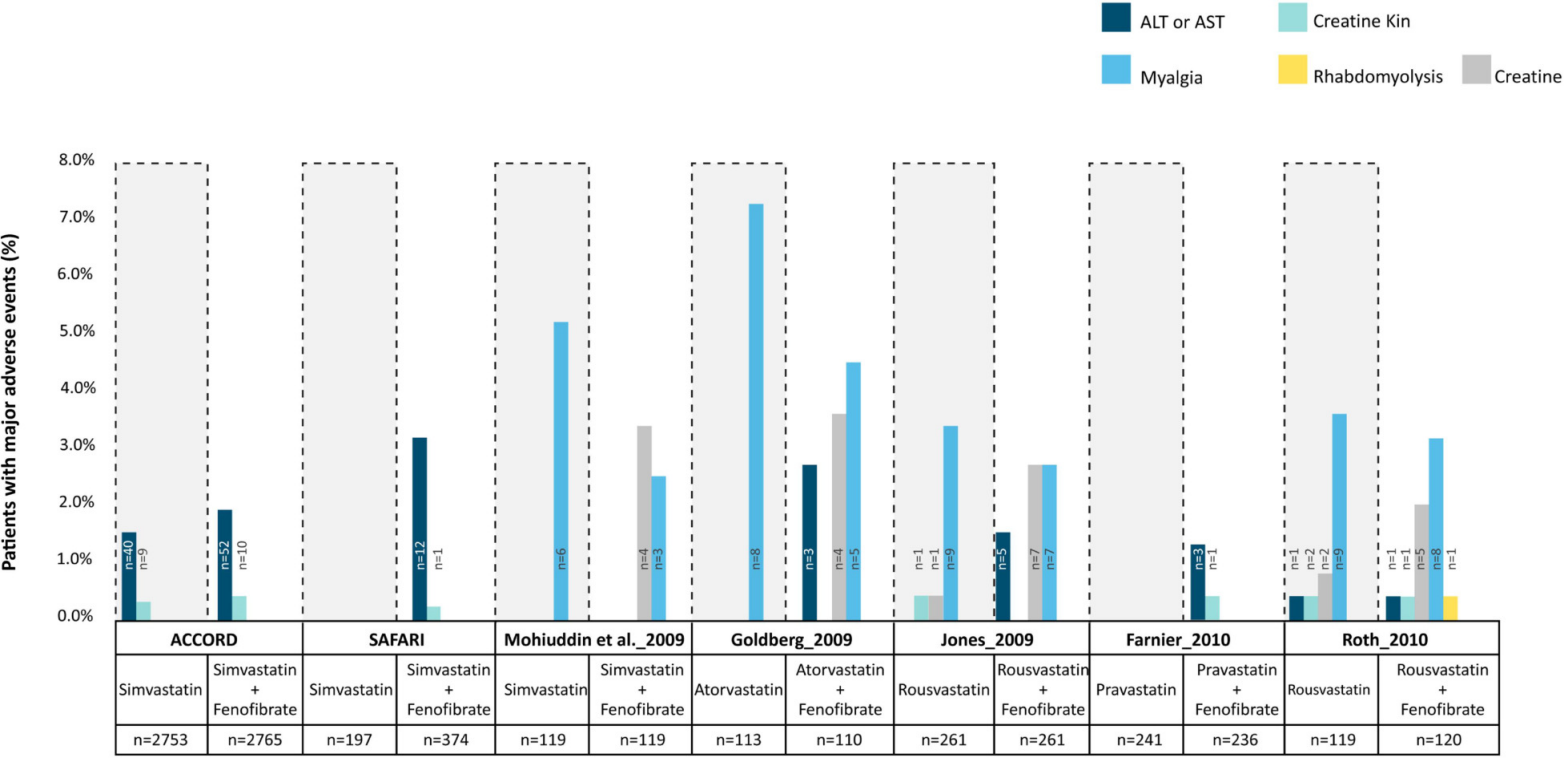
**The safety profile of fenofibrate treatment is comparable to placebo in most studies.** Bar diagram depicting the percentage of major adverse events (***n*** = number of events) in statin monotherapy and fenofibrate-statin combination therapy in the respective studies. For the liver enzymes (ALT or AST) 3× above the upper limit and for kidney/muscle parameters (creatinine and creatinine kinase) 5−10 × upper limit was reported as the adverse event. The total number of patients in each treatment arm is mentioned **(n)** in the table. ALT, alanine aminotransferase; AST, aspartate transaminase. Studies considered for this scatter plot are: ACCORD (48); SAFARI (47); Mohiuddin et al., 2009 (84); Jones et al., 2009 (80); Goldberg et al., 2009 (81); Roth et al., 2010 (82); Farnier et al., 2010 (83).The description of the studies and values for safety parameters depicted in the graph are presented in detail in Supplementary Table S2.

Safety concerns for fibrates in general, mostly based on evidence for other fibrate molecules, have clouded the objectivity of healthcare practitioners in prescribing fenofibrate for long-term use. Evidence from multiple studies confirms that fenofibrate is a well-tolerated fibrate moiety with fewer adverse events as compared to other fibrates, and has a safety profile that is deemed satisfactory as compared to statin monotherapy (70) (Figure 4 and Supplementary Table S2). A meta-analysis of 12 clinical trials reporting data on 5,398 patients concluded that there was no significant difference in adverse events (*P* = 0.54), serious adverse events (*P* = 0.55), drug-related adverse events (*P* = 0.39), or muscle-related adverse events on fenofibrate-statin combinatorial therapy as compared to statin monotherapy (71). Similarly, another meta-analysis of 6 studies using data from 1,628 patients revealed no significant difference in adverse events (2.0% vs 1.5%, *P* = 0.71) in combination vs. monotherapy (72).

Fenofibrate has a lower potential for drug interactions and does not inhibit cytochrome P450 isoenzymes (CYP3A4, CYP2D6, CYP2E1, or CYP1A2) in *in vitro* studies. Fenofibrate does not have a clinically significant impact on the pharmacokinetics of most statins in clinical use (44, 73). Creatine phosphokinase levels do get slightly elevated upon fenofibrate-statin combination treatment (Figure 4 and Supplementary Table S2). However, the concerns about the risk of myopathies due to fenofibrate-statin coadministration were negated in the ACCORD trial. The study reported a similar number of incidences in both intervention and control groups for muscle symptoms (40.1 vs 40.5%), elevation in serum phosphokinase (0.04 vs 0.07%), elevation in serum phosphokinase 10× upper limit (0.4 vs o.3%), and myositis (0.1% in both) (48, 74, 75).

The other adverse events that are generally reported in fenofibrate-statin combinatorial therapy are elevated liver aminotransferases (ALT/AST) (73, 75) (Figure 4 and Supplementary Table S2). Elevation in the levels of hepatic enzymes can occur either within short-term use or after years of latency (occurrence rate: 2%–3% of patients). Even though the incidence of hepatic adverse events is higher in the fenofibrate-statin combination, any serious adverse hepatic event has rarely been observed. Moreover, the elevated hepatic aminotransferase levels are shown to revert to normal in most cases upon therapy termination (48, 74).

While PPAR*α* agonists are known to slightly elevate serum creatinine levels, the latter revert to normal with therapy termination, and fenofibrate usage is not known to cause renal failure or haemodialysis (as seen in the ACCORD) (48, 76). The ACCORDION study reported an increase in the hazard ratio for a composite of adverse renal events (1.16; 95% CI, 1.06 to 1.27) upon fenofibrate-statin usage, but it was primarily driven by incidences of creatinine doubling. However, the doubling of creatinine levels was not confirmed by a repeat test due to study limitations, leading to inconclusive evidence for renal effects with fenofibrate-statin therapy (55, 77).

The PROMINENT trial using pemafibrate in combination with statin reported slightly higher venous thromboembolic events (HR: 2.05; 95% CI, 1.35–3.17; *P* < 0.001) in the intervention arm as compared to the control (61). However, the ACCORD study has not reported any incidence of deep venous thrombosis or pulmonary embolism (48).

Significantly, two real-world studies on a large number of patients with long-term fenofibrate usage (15–20 years), either alone or in combination with statin, have also reported significant improvement in lipids with minimal adverse events (78, 79). Nevertheless, these adverse events are generally mitigated by close monitoring of hepatic and renal parameters. It is also advised to restrict fenofibrate usage in patients with pre-existing hepatic or renal conditions (44).

Overall, fenofibrate is a generally well-tolerated drug and has a good safety profile in combination with statins. The adverse changes in certain biochemical markers are shown to be reversible after treatment cessation, even upon prolonged usage, indicating the absence of irreversible tissue damage.

## 8 Conclusion

The rising global incidence of T2D, obesity, and metabolic disorders has led to an expanding population of patients presenting with elevated levels of TG, TRL, and RC. These individuals are at heightened risk for ASCVD and often require therapeutic strategies that extend beyond standard guideline-directed statin therapy. Despite intensive statin treatment and achievement of target LDL-C levels, a considerable residual CV risk persists in this population.

Emerging evidence has demonstrated that TRLs and RC possess atherogenic potential comparable to that of LDL-C, including their capacity to penetrate the endothelium and contribute to atherosclerotic plaque formation. Given that statins exert modest effects on TG and TRL levels, additional pharmacological strategies in addition to statins are important to comprehensively mitigate the residual ASCVD risk.

Although large cardiovascular outcomes trials evaluating the fenofibrate–statin combination have yielded neutral overall results, prespecified subgroup analyses in patients with elevated triglyceride levels suggest potential benefits of the combination therapy in reducing RC-related residual risk. These findings should be interpreted with caution, given the inherent limitations of subgroup analyses, including reduced statistical power. Confirmation through adequately powered, prospective clinical trials in these specific subgroups remains necessary. In the interim, real-world evidence may offer supportive insights into the potential utility of using fenofibrate-statin combination for addressing residual cardiovascular risk.

Importantly, evidence from trials of TG/TRL-targeted therapies suggests that the CV benefit is more closely associated with reductions in non–HDL-C and ApoB than with TG-lowering *per se*. In this context, the neutral results of the PROMINENT, evaluating a selective PPAR*α* modulator in the high TG-cohort, underscore the importance of reducing overall ApoB-carrying atherogenic particles, rather than just lowering TG/TRL levels. Similarly, trials of other triglyceride-lowering therapies, such as long-chain omega-3 fatty acids (STRENGTH and REDUCE-IT), have demonstrated that clinical benefit is not consistently observed unless accompanied by reductions in non–HDL-C and ApoB, irrespective of their effects on TG levels. Within this framework, fenofibrate-statin combination therapy has been shown to reduce both non–HDL-C and ApoB levels, in addition to lowering TG and TRLs, suggesting a more comprehensive approach to addressing residual CVD risk.

Fenofibrate in combination with statins has a well-documented efficacy and safety profile compared to other triglyceride-lowering therapies, with additional benefits reported for microvascular complications among patients with diabetes. The real-world observational studies have further addressed concerns regarding its long-term use—an essential consideration for chronic combination therapy – demonstrating safety over follow-up durations of up to 15–20 years.

Taken together, management of dyslipidemia should remain patient-centric, taking into account an individual baseline characteristics, presence of comorbidities, drug tolerability, and overall CVD risk, which may restrict uniform applicability of guideline-recommended treatments. Future research should focus on better defining patient phenotypes, most likely to benefit from the fenofibrate-statin combination therapy.
