# Supplementary material for: Managing remnant cholesterol: role of fenofibrate–statin therapy in reducing triglyceride-rich lipoproteins
Source: Front Cardiovasc Med. 2026 Jun 4;13:1837004. doi: 10.3389/fcvm.2026.1837004 (PMC13275287; doi:10.3389/fcvm.2026.1837004)
Supplement: Supplementary file 1 [file Datasheet1.docx]

## Supplementary Figure 1


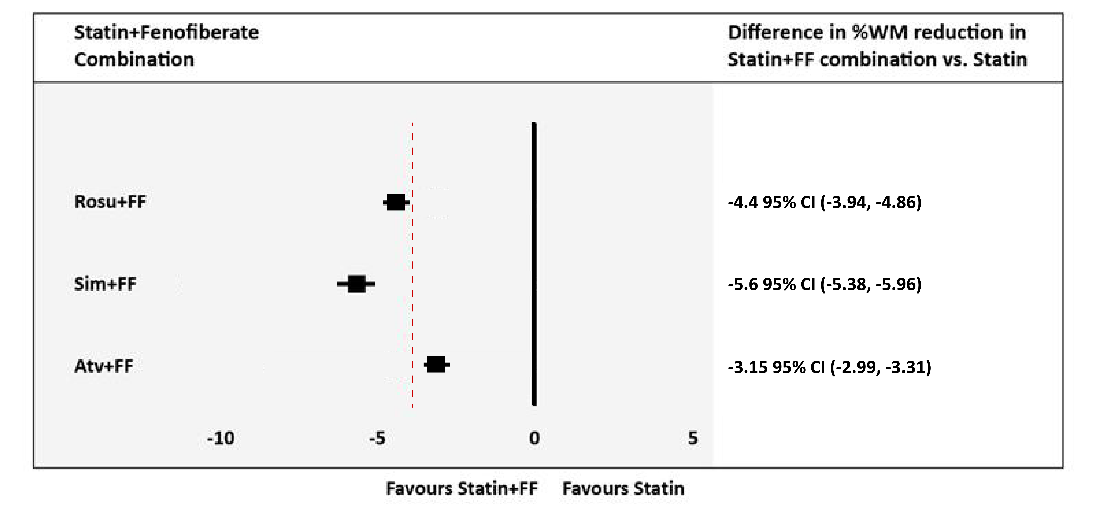


**Supplementary Figure 1:** Fenofibrate-statin combination is efficacious in reducing ApoB levels. The Forest plots represent the difference in percent weighted mean change for ApoB levels in fenofibrate-statin combination and statin monotherapy. **Atv+FF:** Atorvastatin + Fenofibrate; **Sim+FF:** Simvastatin + Fenofibrate; **Rosu+FF:** Rosuvastatin + Fenofibrate; **%WM**: Percent weighted mean

Note: The list of studies and values for ApoB levels used to calculate the percent weighted mean change is presented in Supplementary Table 2

## Supplementary Table 3: Studies used for creating the forest plot and values for the respective lipid parameters

| **Author_year** | **Therapy details** | **N** | **Statin monotherapy** | | | **Statin + Fenofibrate combination** | | | **% mean change in combination vs. monotherapy** | | |
| --- | --- | --- | --- | --- | --- | --- | --- | --- | --- | --- | --- |
|  |  |  | **%non-HDL-C change** | **%RC change** | **% ApoB Change** | **%non-HDL-C change** | **%RC change** | **% ApoB Change** | **% non-HDL-C** | **% RC** | **% ApoB** |
| Davidson  2009 (7) | Atorvastatin 40mg AND Atorvastatin 40mg + Fenofibrate 145mg | **74** | -40.2 ; (P<0.001) | -34.4; (P<0.001) | -35.7 ; (P < 0.001) | -44.8 ; (P < 0.001) | -55.3 (P<0.001) | -40.5 ; (P < 0.001) | -4.6 | -20.8 | -4.8 |
| Goldberg  2009a (8) | Atorvastatin 20mg AND Atorvastatin 20mg + Fenofibrate 135mg | **113** | -35.7 ; (P=0.026) | -24.09 (P<0.001) | -32.9 ; (0.046) | -40.8 ; (P=0.026) | -49.08 (P<0.001) | -37.0 ; (P= 0.046) | -5.1 | -24.9 | -1.8 |
| Goldberg  2009b (8) | Atorvastatin 40mg AND Atorvastatin 40mg + Fenofibrate 135mg | **109** | -41.7 ; (P= 0.001) | -22.2 (P<0.001) | -35.3 ; (P=0.383) | -42.5 ; (P= 0.001) | -49.9 (P<0.001) | -37.1 ; (P=0.383) | -0.8 | -27.8 | -4.1 |
| Athyros  2002 (9) | Atorvastatin 20mg AND Atorvastatin 20mg + Fenofibrate 200mg | **40** | - | -29.8 (P<0.0001) | -31 ; (P < 0.0001) | - | -50 (P<0.0001) | -41 ; (P < 0.0001) | - | -20.1 | -10 |
| Koh  2005 (10) | Atorvastatin 10mg AND Atorvastatin 10mg + Fenofibrate 200mg | **18** | - | -24.9 (P<0.001) | -30.3 ; (P<0.001) | - | -54.1 (P<0.001) | -29.9 ; (P<0.001) | - | -32.2 | -0.4 |
| Lella  2013 (11) | Atorvastatin 10mg AND Atorvastatin 10mg + Fenofibrate 145mg | **30** | - | -19.9 (P<0.001) | - | - | -38.9 (P<0.001) | - | - | -19 | - |
|  |  |  |  |  |  |  |  |  |  |  |  |
| Derosa  2009 (12) | Simvastatin 40 mg/day AND Simvastatin 40 mg/day + Fenofibrate 145 mg/day | **74** | -27.3 ; (P<0.05) | -23.4  (P<0.05) | -16.5 ; (P<0.05) | -41.2 ; (P<0.001) | 38.8  (P<0.001) | -28.7 ; (P<0.01) | -13.9 | -15.4 | -12.2 |
| Foucher  2015 (13)a | Simvastatin 40mg AND Simvastatin 40mg + Fenofibrate 135mg | **112** | -8.7 ; (P=0.003) | -2.5  (P<0.001) | -4.5 ; (P<0.001) | -13.7 ; (P=0.003) | -32.9  (P<0.001) | -9.4 ; (P<0.001) | -5 | -30.4 | -4.9 |
| Mohiuddin  2009a (4) | Simvastatin 20mg AND Simvastatin 20mg + Fenofibrate 135mg | **119** | −24.4 ; (P<0.001) | -29.5  (P<0.001) | −22.9 ; (P<0.001) | −30.7 ; (P<0.001) | -50  (P<0.001) | −29.5 ; (P<0.001) | -6.3 | -20.4 | -6.6 |
| Mohiuddin  2009b (4) | Simvastatin 40mg AND Simvastatin 40mg + Fenofibrate 135mg | **116** | −35.9 ; (P= 0.654) | -43.1  (P<0.001) | -32.7 ; (P= 0.445) | −35.0 ; (P=0.654) | -55.7  (P<0.001) | -31.2 ; (P= 0.445) | 0.9 | -12.5 | -1.5 |
| Krysiak  2011 (14) | Simvastatin 40mg AND Simvastatin 40mg + Fenofibrate 200mg | **49** | - | -24.5  (P<0.05) | -32.0 ; (P < 0.001) | - | -48.1  (P<0.001) | -35.8 ; (P<0.001) | - | -23.8 | -3.8 |
| Foucher  2015b (13) | Simvastatin 20mg AND Simvastatin 20mg + Fenofibrate 135mg | **114** | 0.4 ; (P=0.003) | 0 | -1.1 ; (P<0.001) | -7.9 ; (P=0.003) | -38.5  (P<0.001) | -7.3 ; (P<0.001) | -7.5 | -38.5 | -6.2 |
|  |  |  |  |  |  |  |  |  |  |  |  |
| Jones  2009a (15) | Rousvastatin 20mg AND Rousvastatin 20mg + Fenofibrate 135mg | **266** | −45.8 ; (P<0.001) | -33  (P<0.001) | −39.6 ; (P=0.729) | -45.3 ; (P<0.001) | -50.2  (P<0.001) | −39.2 ; (P=0.729) | -0.5 | -17.2 | -0.4 |
| Roth  2010 (5) | Rosuvastatin 5mg AND Rosuvastatin 5mg + Fenofibric acid 135 mg. | **241** | –31.8 ; (P<0.001) | -27.7  (P<0.001) | -26.4 ; (P=0.001) | –37.4 ; (P<0.001) | -46.9  (P<0.001) | -30.9 ; (P=0.001) | -5.6 | -19.3 | -4.5 |
| Jones  2009b (15) | Rousvastatin 10mg AND Rousvastatin 10mg + Fenofibrate 135mg | **261** | −39.8 ; (P<0.001) | -31.5  (P<0.001) | −34.1 ; (P<0.001) | -44.7 ; (P<0.001) | -49.9  (P<0.001) | -39.2 ; (P<0.001) | -4.9 | -18.4 | -5.1 |
| Durrington  2004 (16) | Rousvastatin 10mg AND Rosuvastatin 10 mg + Fenofibric acid 135 mg | **51** | - | -28.3  (P<0.001) | -41.4* | - | -47.1* | -40.2* | - | -18.8 | 1.2 |

**P* value not available in the study; %, percent; ApoB, apolipoprotein B; non-HDL-C, non-high density lipoprotein cholesterol; RC, remnant cholesterol

## Methods S1

Studies using equal dosages of statins in the intervention and comparator arms were selected. The pooled estimate across drug combinations was obtained using a meta-analysis framework. Each statin–fenofibrate combination was treated as an individual study, and effect sizes (mean relative change with standard errors) were calculated as described below.

A weighted mean ($\bar{X}_{w}$) of the relative change ($C$) in the respective lipid parameter was calculated using patient numbers ($N$) as weights using the following formula:

$$\bar{X}_{w}=\frac{\sum\left( N_{i}\times C_{i} \right)}{\sum Nⅈ}$$

The weighted variance ($s_{w})$was computed as:

$$s_{\omega}^{2}=\frac{\Sigma N_{i}\left( C_{i}-\bar{X}_{w} \right)}{\Sigma N_{i}}$$

The standard error (SE) of the weighted mean was then calculated as:

$$SE=\frac{s_{w}}{\sqrt{\Sigma N}ⅈ}$$

A 95% confidence interval (CI) for the weighted mean was derived using a normal approximation:

95% CI=$\bar{X}_{w}$±1.96×SE

The analyses were conducted using standard meta-analysis formulas, and results were presented using a forest plot to display the weighted mean change in the respective lipid parameter with 95% CIs.

## References

1. Das Pradhan A, Glynn RJ, Fruchart JC, MacFadyen JG, Zaharris ES, Everett BM, et al. Triglyceride Lowering with Pemafibrate to Reduce Cardiovascular Risk. N Engl J Med. 2022;387(21):1923-34.

2. Arai H, Yamashita S, Yokote K, Araki E, Suganami H, Ishibashi S, et al. Efficacy and safety of K-877, a novel selective peroxisome proliferator-activated receptor alpha modulator (SPPARMalpha), in combination with statin treatment: Two randomised, double-blind, placebo-controlled clinical trials in patients with dyslipidaemia. Atherosclerosis. 2017;261:144-52.

3. Grundy SM, Vega GL, Yuan Z, Battisti WP, Brady WE, Palmisano J. Effectiveness and tolerability of simvastatin plus fenofibrate for combined hyperlipidemia (the SAFARI trial). Am J Cardiol. 2005;95(4):462-8.

4. Mohiuddin SM, Pepine CJ, Kelly MT, Buttler SM, Setze CM, Sleep DJ, et al. Efficacy and safety of ABT-335 (fenofibric acid) in combination with simvastatin in patients with mixed dyslipidemia: a phase 3, randomized, controlled study. Am Heart J. 2009;157(1):195-203.

5. Roth EM, Rosenson RS, Carlson DM, Fukumoto SM, Setze CM, Blasetto JW, et al. Efficacy and safety of rosuvastatin 5 mg in combination with fenofibric acid 135 mg in patients with mixed dyslipidemia - a phase 3 study. Cardiovasc Drugs Ther. 2010;24(5-6):421-8.

6. Farnier M, Ducobu J, Bryniarski L. Efficacy and safety of adding fenofibrate 160 mg in high-risk patients with mixed hyperlipidemia not controlled by pravastatin 40 mg monotherapy. Am J Cardiol. 2010;106(6):787-92.

7. Davidson MH, Rooney MW, Drucker J, Eugene Griffin H, Oosman S, Beckert M, et al. Efficacy and tolerability of atorvastatin/fenofibrate fixed-dose combination tablet compared with atorvastatin and fenofibrate monotherapies in patients with dyslipidemia: a 12-week, multicenter, double-blind, randomized, parallel-group study. Clin Ther. 2009;31(12):2824-38.

8. Goldberg AC, Bays HE, Ballantyne CM, Kelly MT, Buttler SM, Setze CM, et al. Efficacy and safety of ABT-335 (fenofibric acid) in combination with atorvastatin in patients with mixed dyslipidemia. Am J Cardiol. 2009;103(4):515-22.

9. Athyros VG, Papageorgiou AA, Athyrou VV, Demitriadis DS, Kontopoulos AG. Atorvastatin and micronized fenofibrate alone and in combination in type 2 diabetes with combined hyperlipidemia. Diabetes Care. 2002;25(7):1198-202.

10. Koh KK, Quon MJ, Han SH, Chung WJ, Ahn JY, Seo YH, et al. Additive beneficial effects of fenofibrate combined with atorvastatin in the treatment of combined hyperlipidemia. J Am Coll Cardiol. 2005;45(10):1649-53.

11. Lella M, Indira K. A comparative study of efficacy of atorvastatin alone and its combination with fenofibrate on lipid profile in type 2 diabetes mellitus patients with hyperlipidemia. J Adv Pharm Technol Res. 2013;4(3):166-70.

12. Derosa G, Maffioli P, Salvadeo SA, Ferrari I, Gravina A, Mereu R, et al. Fenofibrate, simvastatin and their combination in the management of dyslipidaemia in type 2 diabetic patients. Curr Med Res Opin. 2009;25(8):1973-83.

13. Foucher C, Aubonnet P, Reichert P, Berli M, Schaeffer A, Calvo Vargas CG, et al. New Fixed-Dose Combinations of Fenofibrate/Simvastatin Therapy Significantly Improve the Lipid Profile of High-Risk Patients with Mixed Dyslipidemia Versus Monotherapies. Cardiovasc Ther. 2015;33(6):329-37.

14. Krysiak R, Gdula-Dymek A, Okopien B. Effect of simvastatin and fenofibrate on cytokine release and systemic inflammation in type 2 diabetes mellitus with mixed dyslipidemia. Am J Cardiol. 2011;107(7):1010-8 e1.

15. Jones PH, Davidson MH, Kashyap ML, Kelly MT, Buttler SM, Setze CM, et al. Efficacy and safety of ABT-335 (fenofibric acid) in combination with rosuvastatin in patients with mixed dyslipidemia: a phase 3 study. Atherosclerosis. 2009;204(1):208-15.

16. Durrington PN, Tuomilehto J, Hamann A, Kallend D, Smith K. Rosuvastatin and fenofibrate alone and in combination in type 2 diabetes patients with combined hyperlipidaemia. Diabetes Res Clin Pract. 2004;64(2):137-51.
